# ADDRESSING SOCIAL DETERMINANTS OF HEALTH DURING CARE TRANSITIONS: AN INTERDISCIPLINARY PERSPECTIVE

**DOI:** 10.1093/geroni/igae098.1421

**Published:** 2024-12-31

**Authors:** Ayomide Bankole

**Affiliations:** Chair

## Abstract

Care transitions, such as transitions from hospital to home or home health care, are particularly challenging periods for many older adults with unaddressed social determinants of health (SDOH) needs. For instance, older adults might experience barriers in accessing transportation for follow-up medical appointments or acquiring prescribed medications due to financial limitations. These challenges significantly impact health outcomes, often resulting in emergency department visits and re-hospitalizations for many older adults. Addressing social determinants during care transitions is crucial for promoting safe, effective, equitable, and quality healthcare delivery, particularly for those with the greatest social vulnerabilities. In this symposium, we present research, and recommendations from an interdisciplinary group of clinicians from nursing, rehabilitation therapy, and social work disciplines. Dr. Osokpo will start the symposium by proposing a theoretical framework for addressing social determinants during care transitions. Dr. Bankole will describe the characteristics of socio-economically disadvantaged older adults (dual eligible beneficiaries) who transition to home healthcare, for post-acute care services. Dr. Nguyen will discuss the implementation and evaluation of a transitional care intervention that addresses service needs for dementia caregivers and those whose primary language is not English. For the final presentation, Denise Dews MSW will describe an interprofessional collaborative approach to delivering high-quality transitional care to improve outcomes for socially vulnerable older adults. Together, these presentations will offer recommendations to enhance care transitions, with implications for interprofessional clinical education, practice, and research.

